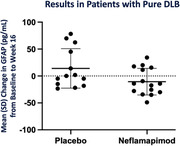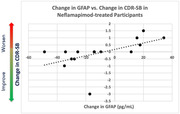# Neflamapimod treatment reduces plasma glial fibriallary acidic protein levels in patients with pure dementia with Lewy bodies (DLB)

**DOI:** 10.1002/alz.091713

**Published:** 2025-01-09

**Authors:** John J Alam, Marleen JA Koel‐Simmerlink, Jennifer Conway, Inge M.W. Verberk, Charlotte Teunissen

**Affiliations:** ^1^ CervoMed, Boston, MA USA; ^2^ Amsterdam UMC, Amsterdam Netherlands; ^3^ Neurochemistry Laboratory, Department of Clinical Chemistry, Amsterdam Neuroscience, Vrije Universiteit Amsterdam, Amsterdam UMC, Amsterdam Netherlands; ^4^ Neurochemistry Laboratory, Department of Laboratory Medicine, Amsterdam Neuroscience, Amsterdam UMC, Vrije Universiteit Amsterdam, Amsterdam Netherlands

## Abstract

**Background:**

In a 16‐week, 91‐patient placebo‐controlled clinical study in DLB (“AscenD‐LB”;NCT04001517), neflamapimod improved outcomes on the CDR Sum‐of‐Boxes (p = 0.023 vs. placebo) and Timed Up and Go test (p = 0.044 vs. placebo). Further, at the higher of two dose levels in the study neflamapimod also improved (p = 0.049 vs. placebo) outcomes in a cognitive test battery, particularly with respect to attention (p = 0.023 vs. placebo). The results were most prominent in patients with pure DLB (i.e., without Alzheimer’s disease‐related co‐pathology, assessed by plasma ptau181). While no validated biomarkers for treatment effects in DLB were available when the study was conducted, with a recent report that plasma GFAP and neurofilament‐light‐chain (NfL) discriminated MCI‐DLB from healthy controls, with GFAP being more discriminant, we evaluated the effects of neflamapimod in AscenD‐LB on plasma GFAP and NfL, both overall and in pure DLB patients.

**Method:**

GFAP and NfL levels (pg/mL) in stored plasma samples from AscenD‐LB were determined using Simoa® platform. Design and overall results (Jiang et.al. Nature Communications, 2022), and analysis after stratification for baseline plasma ptau181 (Alam et.al. Neurology, 2023) are published.

**Result:**

At baseline, GFAP was higher (p = 0.02) in DLB with AD co‐pathology [282(SD = 120, n = 29] versus in pure DLB [215(SD = 91), n = 28]; NfL was not. In the overall population, a reduction in GFAP levels were seen in neflamapimod‐treated participants (n = 30, ‐12.3±6.8, baseline‐to‐week16) and not in placebo‐recipients (n = 27, +3.7±7.8; p = 0.13); p = 0.13 for the difference. In pure DLB participants, the change from baseline to week 16 in GFAP was significantly different (p = 0.04) in neflamapimod‐treated participants (n = 15, ‐10.6±6.4) compared to placebo‐recipients (n = 13, +14.1±10.2). For NfL, no neflamapimod‐placebo differences were evident in either population. In the pure DLB population, the change in GFAP levels from baseline was significantly correlated to change in CDR‐SB (r = 0.54, p = 0.036; increased GFAP associated with worsening CDR‐SB, reduction in GFAP associated with improvement on CDR‐SB) among neflamapimod recipients, but not in placebo‐recipients.

**Conclusion:**

The beneficial effects on GFAP further support that neflamapimod is clinically efficacious in patients with DLB. The results also demonstrate the potential of GFAP as biomarker to assess treatment effects in DLB, though treatment effects may be masked in patients with AD co‐pathology.